# Pleasant touch perception in borderline personality disorder and its relationship with disturbed body representation

**DOI:** 10.1186/s40479-021-00176-4

**Published:** 2022-02-01

**Authors:** Annette Löffler, Nikolaus Kleindienst, Corinne Neukel, Robin Bekrater-Bodmann, Herta Flor

**Affiliations:** 1 Institute of Cognitive and Clinical Neuroscience, Central Institute of Mental Health, Medical Faculty Mannheim, Heidelberg University, Square J5, 68159 Mannheim, Germany; 2 Institute of Psychiatric and Psychosomatic Psychotherapy, Central Institute of Mental Health, Medical Faculty Mannheim, Heidelberg University, Mannheim, Germany; 3 Department of General Psychiatry, Centre for Psychosocial Medicine, Heidelberg University, Heidelberg, Germany; 4 Department of Psychosomatic Medicine and Psychotherapy, Central Institute of Mental Health, Medical Faculty Mannheim, Heidelberg University, Mannheim, Germany

**Keywords:** Pleasant touch, Borderline personality disorder, Affective startle modulation, Body representation, Dissociation

## Abstract

**Background:**

Borderline personality disorder (BPD) is characterized by altered perception of affective stimuli, including abnormal evaluation of nociceptive input. However, whether or not perceptual alterations are present for its positive counterpart, i.e. pleasant touch (PT), has not yet been examined.

**Methods:**

In the present study, we applied standardized PT stimuli to the hands of 25 patients with BPD and 25 healthy controls (HC) and compared their perception. We used the affect-modulated acoustic startle response as a physiological correlate of affective processing. We further explored the effect of PT stimulation on dissociative experiences in BPD.

**Results:**

Compared to HC, BPD perceived PT as less pleasant and less intense. The effect on perceived valence of touch was large even after controlling for the effect of reduced perceived intensity of touch (*ƞ*^*2*^ = .29). We further found qualitative alterations in touch perception in BPD, who rated the touch as significantly rougher and firmer compared to HC. There was a positive correlation between perceived valence of touch and changes in dissociative experiences in terms of body ownership of the stimulated body part from pre to post stimulation, suggesting that a more negative evaluation of touch is associated with an increase in body-related dissociative experiences, while a positive perception of touch might be related to a reduction of these dissociative experiences.

**Conclusions:**

Our results confirm BPD-associated disturbances in the processing of affective somatosensory stimuli and indicate that not only pain perception but also pleasant touch perception is diminished in BPD. We discuss the role of altered touch perception for BPD psychopathology and its potential role for new treatment approaches.

**Supplementary Information:**

The online version contains supplementary material available at 10.1186/s40479-021-00176-4.

## Background

Borderline personality disorder (BPD) is a severe mental disorder characterized by dysfunctional affect regulation, impulsivity, problematic social interaction, and an unstable self-image [1]. All of these features are related to altered processing of affective stimuli, with evidence supporting the assumption of a negativity bias [2]. Altered affective processing has mainly been shown for the responses to negatively valenced stimuli [3]. But there is growing evidence that positive affective processing is altered as well [4]. In comparison to healthy controls (HC), negative evaluation tendencies have been reported for positive facial expressions [e.g. 5, 6], positive taste stimuli [7], and positive social cues in terms of self-relevant appreciating sentences [8].

Whereas previous studies repeatedly demonstrated significant reductions in pain sensitivity [9–15], with the affective component of pain, compared to its sensory-discriminative component, being particularly affected [e.g. 16], they found no evidence for altered proprioception, exteroception, and two-point discrimination [17] or warmth perception thresholds [9, 10,c.f. 18], suggesting that somatosensory dysfunction is limited to affective stimuli. However, it remains unclearwhether altered somatosensory processing in BPD is specific for pain perception or also affects positive affective somatosensation.

A recent systematic review and meta-analysis proposes light stroking at a velocity of approximately 3 cm/s for the assessment of somatosensory pleasantness, i.e. pleasant touch, parallel to the assessment of pain [19]. Thereby, C-tactile (CT) afferents, which are activated by touches applied with velocities between 1 and 10 cm/s, seem to mainly code pleasant sensation [20, 21]. In addition, other types of afferents might contribute, as stroking touch can be pleasant when applied to glabrous skin, a site lacking CT afferents [22]. Interestingly, on a central level there is a common neurobiology of pain and pleasure [23] with common involvement of brain regions such as insula, amygdala, prefrontal cortex and orbitofrontal cortex. Due to the neurobiological similarities between pain and pleasure, it can be hypothesized that the processing of both positive and negative affective somatosensory information might be altered in BPD. The assumption of altered pleasant touch processing in BPD is also supported by a previous study by Croy et al. [24], who assessed pleasant touch perception in a heterogeneous sample of psychotherapy outpatients suffering from different disorders (mood and affective disorders, post traumatic stress disorder, anxiety disorder, personality disorders). In this study, patients, particularly those with personality disorders, rated pleasant touch as less pleasant compared to HC. However, Croy et al. [24] did not report results for different types of personality disorders, so that BPD-associated alterations remain unknown.

To assess positive and negative affective processing on a physiological level, the affect-modulated acoustic startle response is a common peripheral physiological measure. The magnitude of the blink response to a startling acoustic probe is increased when unpleasant stimuli are processed and decreased during pleasant stimulation [25]. A recent study confirmed that the response strength is modulated primarily by the centromedial region of the amygdala [26], while the prefrontal cortex has shown to play an important role specifically in pleasure-induced inhibition of the startle response [27]. Amygdala and prefrontal dysfunctions have been identified as important neural deficits in BPD [28] and have been further related to processing of affective stimuli in the disorder [3]. In line with this, previous studies assessing affect-modulated startle responses in BPD found exaggerated affective startle in response to negative and borderline-salient stimuli compared to HCs [29, 30,but see 31]. Therefore, dampened affect-modulated acoustic startle responses might serve as a physiological correlate for less pleasant processing of pleasant touch in BPD.

Experimental studies in healthy subjects suggest that pleasant touch might play a role in the experience of body ownership, i.e. the sensation that the body and all its parts belong to oneself. Compared to neutral touch, pleasant touch has shown to produce higher levels of ownership for an artificial limb in the rubber hand illusion paradigm [e.g. 32] and can reduce the feeling of deafference [33], i.e., unpleasant and numbness sensation about the body induced by a temporal mismatch between seen and felt tactile stimulation [34]. Moreover, a recent study on neurological patients with reduced body ownership indicates that the application of pleasant touch could increase body ownership experiences [35]. Interestingly, dissociation, a common symptom and diagnostic feature of BPD [36], includes the feeling of foreignness related to the own body, and body ownership experiences have been shown to be reduced in BPD [37]. Thereby, from a psychopathological perspective, it might be interesting to assess whether pleasant touch stimulation might modulate (dissociative) body experiences in BPD.

The main aim of the present study was to investigate whether perception of positive somatosensory stimulation is less positive in BPD compared to HC. Therefore, we applied standardized pleasant touch to the back of the hand of participants with BPD and a sample of HC. We specifically expected a less positive perception of pleasant touch assessed by self-report in BPD compared to HC. On a physiological level, we expected a diminished inhibition of the acoustic startle response in the BPD versus control group. In order to investigate the specificity of somatosensory alterations for affective stimuli, we assessed mechanical and warm perception as well as heat pain thresholds of the skin. Thereby, we expected to replicate heightened pain thresholds in BPD compared to HC, and further expected an association between deficient processing of pleasant touch and deficient pain processing. Specifically a more negative evaluation of pleasant touch was assumed to be associated with higher levels of heat pain thresholds. We further explored whether there is a pleasant touch-associated modulation of dissociation and dissociative body experiences in terms of reduced body ownership in BPD.

## Methods

### Sample

We examined 27 female BPD patients and 26 female healthy controls (HC) who were centrally recruited by Clinical Research Unit 256 [38]. The measurement had to be prematurely terminated due to intolerable tension evoked by the experimental paradigm in two subjects with BPD and circulatory problems in one HC subject. Accordingly, the final sample consisted of 25 subjects with a current diagnosis of BPD (mean (*M*) age = 31.28 years, standard deviation (*SD*) = 7.57) and 25 HC (*M* age = 26.72 years, *SD* = 8.57). The groups did not significantly differ in age, *t* (48) = 2.00, *p* > .05. All subjects were fluent in German and all but three participants were right-handed (two ambidextrous participants in the BPD and one ambidextrous participant in the HC group) as assessed with the Edinburgh Handedness Inventory [39]. Regular psychotropic and pain medication had to be discontinued for at least two weeks prior to study participation (with the exception of selective serotonin reuptake inhibitors (SSRIs), of which discontinuation is not recommended given the evidence for adverse physical and psychological symptoms that may occur with its discontinuation [40]). None of the subjects had been on on-demand medication (such as sedative-hypnotics or benzodiazepines) for two days prior to participation. The study was approved by the Ethics Commission of the Medical Faculty Mannheim of Heidelberg University and complied with the Declaration of Helsinki. All participants provided written informed consent and received a reimbursement of 26€ for participation.

Clinical diagnoses according to the Diagnostic and Statistical Manual for Mental Disorder IV (DSM-IV) [41] were obtained by a trained clinical psychologist using the Structured Clinical Interview for DSM-IV Axis I Disorders (SCID) [42] and the International Personality Disorder Examination (IPDE) [43]. BPD patients had to meet five or more of the BPD IPDE criteria within the last two years prior to study participation, and at least one of these criteria had to be present during childhood or adolescence.

We excluded subjects with scars on the back of the left hand (due to self-injurious behavior or other reasons) to avoid a potential bias due to reduced sensitivity in the stimulated body part. Further exclusion criteria were life-time diagnosis of bipolar I disorder or schizophrenia, insufficient speech comprehension, mental retardation, body mass index < 16.5, substance use disorder within the last year (in case of current substance abuse, abstinence of at least two months was required), fibromyalgia, serious physical illness, severe brain disorder or concussion, and pregnancy. The prevalence of comorbid life-time and current mental disorders as well as psychopathological characteristics of the BPD sample are given in Table 1. No current or life-time mental disorders were present in the HC group, as assessed with the SCID [42].

**Table 1:** Prevalence of comorbid axis I disorders and psychopathological characteristics of the borderline personality disorder sample (*n* = 25)

	Prevalence		Psychopathological characteristics	
	Current ***n*** (%)	Life-time^**1**^ ***n*** (%)		***M (SD)***
**Major depressive disorder**	7 (28)	18 (72)	**Symptom severity (BSL-23)** **(*n*** **= 24)**	1.59 (0.70)
**Post-traumatic stress disorder**	8 (32)	15 (60)	**Depressiveness (BDI)** ***(n =*** **23*)***	18.26 (8.00)
**Anorexia nervosa**	0 (0)	6 (24)	**Trait anxiety (STAI)** ***(n =*** **24*)***	63.29 (6.31)
**Other Eating disorders**	7 (28)	7 (28)	**State anxiety (STAI)** **(*n*** **= 23)**	54.87 (9.30)
**Other mental disorders (only current)**	20 (80)	–		
**More than one mental disorder (only current)**	11 (44)	–		

BPD = borderline personality disorder; *n* = number; *M* = mean; *SD* = standard deviation; BSL-23 = Borderline Symptom List [44], BDI = Beck Depression Inventory [45], STAI = State-Trait-Anxiety Inventory [46]

^1^Including current diagnosis

### Psychological assessment

To assess general symptom severity, we used the mean score of the *Borderline Symptom Lis*t (BSL-23) [44]. Values are ranging from 0 to 4 with higher values indicating a higher symptom severity. To assess depressiveness, we used the *Beck Depression Inventory* (BDI) [45]. The overall sum score ranges from 0 to 63 with higher values indicating higher depressiveness. The *State-Trait-Anxiety Inventory* (STAI) [46] was used to assess anxiety. The sum score for the state and trait subscale, ranges from 40 to 160 each, with higher values indicating higher anxiety. Data of BSL-23, and STAI (trait) were missing for one BPD subject, BDI and STAI (state) data were missing for two BPD subjects.

### Experimental paradigm

**Fig. 1:**
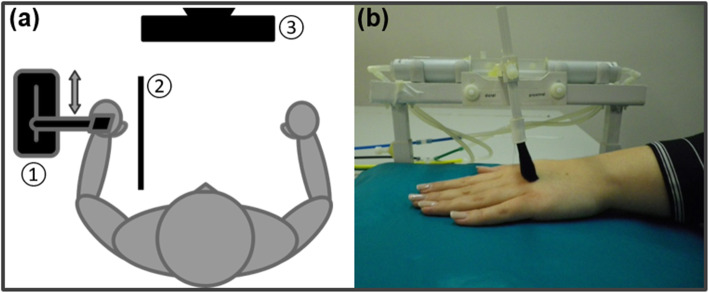
**(a)** Schema of the experimental setting consisting of a brushing machine (1), a privacy screen (2), and a computer screen (3); **(b)** Stimulation of the back of the left hand (i.e., hairy skin) using a soft brush

The experimental setup is depicted in Fig. 1a. We applied pleasant touch stimulation on the subjects’ back of the left hand (i.e., hairy skin) using a soft brush and a custom apparatus (see Fig. 1b), which applied touch without social interaction and with a standardized velocity of 3 cm/s. Using a similar device and setup in HCs, has been shown to validly evoke a pleasant touch percept as well as touch-related activation in brain areas typically involved in the processing of pleasant touch [47]. The left hand of the subjects was placed on a vacuum cushion underneath the brush to rest it comfortably and stably during the experiment. In order to apply gentle touches with comparable forces, the brush was adjusted for each subject dependent on the size of the individual’s hand in a way that just the tip of the brush touched the skin. Using a privacy screen, the pleasant touch device and the stimulated hand were positioned out of the subjects’ view. The subjects were instructed to fixate a cross presented on a screen in front of them and attend to their stimulated left hand throughout the experiment. The touch stimulation was applied in 12 blocks, lasting 60s each, with a stroke length of 8 cm, going back and forth between the metacarpal bones of the participants’ left third and fourth digit.

After each block, the subjects were asked to rate the perceived intensity and valence of touch. Intensity of touch was assessed using a visual analogue scale (VAS) using the verbal anchors “no perception” and “very intense perception”. Perceived pleasantness of touch was assessed with a VAS with the anchors “not pleasant” and “very pleasant”, and perceived unpleasantness of touch was assessed with a separate VAS using the anchors “not unpleasant” and “very unpleasant”. For all VAS scales the answers were converted in values ranging from 0 (“no perception”, “not pleasant”, not unpleasant”) to 100 (“very intense perception”, “very pleasant”, “very unpleasant”). We assessed valence of touch in a two-dimensional manner in accordance with previous recommendations [48–50], and combined the ratings of pleasantness and unpleasantness for the main analyses by subtracting the unpleasantness rating from the pleasantness rating, resulting in a bipolar valence score ranging from − 100 (indicating maximum net negative valence) to + 100 (indicating maximum net positive valence) with values of 0 indicating neutral valence. Furthermore, subjects rated qualitative aspects of touch perception by using a German adaptation of the touch perception task (TPT) [51] (see the supplement for translation, cultural adaptation, and details of the assessment of the TPT items. The TPT assesses touch perception by four empirically identified sensory factors (slip, pile, roughness and firmness) with 26 descriptors and two affective factors (comfort and arousal) with 14 descriptors (see Table S1 in the supplement).

To assess state dissociation before and after touch application, we used the mean score of the short version of the Dissociation-Tension Scale acute (DSS-4) [52], immediately before the experiment started and after the last block of stimulation. Before and after pleasant touch application we further asked participants for perceived body ownership disturbances employing the shortened version of a previously used body ownership interview [37], assessing ownership for the right and left arm. Participants were asked to verbally rate the perceived degree of current ownership for their shoulders, upper and lower arms as well as hands (left and right each) by indicating a percentage from 0% (“body part does not belong to me”) up to 100% (“body part belongs to me”) in 10% increments. To assess disturbances of body ownership in BPD of both the stimulated and non-stimulated arm, we separately computed the mean score of the left (internal consistency in the present study α = .96 before and α = .94 after stimulation) and right (internal consistency in the present study α = .94 before and α = .95 after stimulation) body sites. All items were presented on the computer screen in front of the participants, and were answered using a keyboard. The experiment was programmed in Presentation (v17.0; Neurobehavioral Systems, Inc., Albany, CA, USA).

### Startle data collection and scoring of affect-modulated acoustic startle response

Recording and analysis of startle data followed the recommendations by Blumenthal et al. [53]. A 50 ms white noise burst set to a volume of 95db was used as acoustic startle probe, and eight startle probes were presented for habituation purposes before the experiment started. Experimental probes then occurred randomly once or twice at least 15 s after the onset of touch in half of the stimulation-blocks, with an inter-probe interval of at least 18 s [54]. Stimuli were presented randomly once or twice in half of the fixation-blocks (a fixation-block with a random duration between 60 and 69 s without stimulation preceded each block of touch application and served as baseline interval). The startle probe occurred 9 times with and 9 times without tactile stimulation. The probes were delivered using the amplifier Phone Amp G100 (Lake People, Konstanz, Germany) and insert earphones EarTone® 3A 10-Ohm (Aearo Company Auditory Systems Production, Indianapolis, USA) with 10 mm earplugs (ER3-14B, Etymotic Research Inc., Elk Grove Village, USA). The acoustic startle response was measured by recording electromyographic (EMG) activity of the musculus orbicularis oculi of the left eye using Ag-AgCl electrodes with a diameter of 40 mm filled with high-conductivity electrode gel (Electro Cap International Inc., Eaton, USA). The ground electrode was attached to the right forehead. The electrodes were applied between the threshold assessments and the experiment. The skin was cleaned with alcohol and abraded using V17 Abralyt 2000 (Easycap GmbH, Herrsching, Germany) to lower the impedances. Physiological data were amplified and recorded using a BrainAmp ExG amplifier (Brain Vision, Morrisville, USA) and the Brain Vision Recorder software v1.10 (Brain Vision, Morrisville, USA). The sampling rate was set to 5000 Hz. Frequencies below 28 Hz and above 400 Hz were filtered out, and a notch filter of 50 Hz was applied.

Brain Vision Analyzer v2.0 was used for offline analysis of the EMG signal. Startle amplitude was defined as the difference between the peak startle activity within a time window of 20 ms to 120 ms after stimulus onset and the mean EMG activity 50 ms before stimulus onset. Prior to analysis, EMG recordings were visually examined and screened for artefacts. Segments with noise, movement artefacts or spontaneous or voluntary blink before the minimal onset latency value within a time frame of 50 ms before to 200 ms after stimulus onset, or segments without a startle reaction (defined as peaks below 10 μV) were excluded from analysis. Remaining segments were rectified and smoothed by a moving average with a 10 ms window. Finally, segments of the fixation-block and the stimulation-block were averaged separately. The affect-modulated acoustic startle response (ASR) was calculated in percent by the following formula:

$$ \frac{\left( startle\ during\ stimulation\right)-\left( startle\ without\ stimulation\right)}{\left( startle\ without\ stimulation\right)}\times 100. $$

Thus, positive scores indicate an increase in startle amplitude during stimulation relative to baseline and negative scores indicate a decrease. Reporting relative scores for affect-modulated ASR is recommended to remove any dependence on baseline eye blink amplitude [53]. Due to technical problems during data recording, we could not sample physiological data of three BPD and two HC participants. To ensure that a sufficient number of trials per block were included, the required number of valid segments per subject and block (on/off) was set to four. Using this criterion, we had to exclude additional three BPD and three HC participants. Thus, we analyzed physiological data of 19 BPD and 20 HC subjects.

### Assessment of thresholds for mechanical detection, warm perception, and heat pain

Touch sensitivity was assessed at the beginning of the experiment by mechanical detection thresholds using the standard examination protocol for Quantitative Sensory Testing (QST) of the German Research Network on Neuropathic Pain [55]. Thresholds were recorded on the skin between the metacarpal bones of the third and fourth finger of the participants’ left back of the hand. We used a standardized set of von-Frey filaments with forces between 0.25mN and 512mN (Opti-hair_2_, MARSTOCK-Nervtest, Schriesheim, Germany), implementing a staircase procedure to ascertain the mechanical threshold, defined as the geometric mean of five below- and five above-threshold intensities.

We then assessed warm perception and heat pain thresholds using a Thermal Sensory Analyzer device (Medoc Ltd., Ramat Yishai, Israel) with a 30x30mm thermode attached to the subjects’ left thenar. Starting at a baseline temperature of 32 °C, the temperature rose with a rate of 1.2 °C/s for the assessment of the warm perception threshold, and with a rate of 3 °C/s for the assessment of heat pain threshold [56]. The subjects signaled the onset of warm perception or heat pain perception by pressing a button resulting in a fall of temperature back to baseline temperature in five trials each. The first trial served as familiarization trial while the average of the remaining four trials was used for further analyses. For safety reasons, the thermode was shut down when a temperature of 52 °C was reached. This safety limit was reached in 3 trials of one BPD patient and in one trial of two HC each. For these trials, the temperature was rounded to 53 °C [9]. Due to technical problems with the thermal stimulator during the main period of data assessment, we could not assess thresholds in eleven BPD subjects and one HC, and thus, subsequent analyses on perception thresholds were performed only for subsamples.

### Statistical analyses

Data were tested for normal distribution using a Kolmogorov-Smirnov test. If the assumption of normality was violated, non-parametric statistics were used. To test our main hypothesis, we compared data of BPD and HC participants for perceived valence, intensity, and qualitative aspects of pleasant touch using t-tests for independent samples or, in the case of non-normal distribution, Mann-Whitney-U-Tests. Because there was an unexpected difference in perceived intensity of touch between BPD and HC, a robust rank based ANCOVA [57] was performed to control for perceived intensity of touch on the effects of perceived valance of touch. We further correlated perceived valence and intensity of touch with symptom severity as assessed by the BSL in the BPD group using Pearson or Spearman correlations. Additionally, we compared both groups regarding affect-modulated ASR, and correlated affect-modulated ASR with perceived valence and intensity of touch using Pearson or Spearman correlation in both groups separately.

To explore the effect of pleasant touch stimulation on dissociation in BPD, we compared state dissociation and arm ownership before and after pleasant touch stimulation using paired t-tests or, in the case of non-normal distribution, Wilcoxon signed-rank tests. Further, we calculated difference scores in state dissociation and arm ownership from before to after pleasant touch stimulation. We correlated perceived valence and intensity of touch with the change in dissociation and arm ownership of the stimulated and non-stimulated arm using Spearman rank correlations. In order to test whether there was a specific effect for the correlation between perceived valence of touch and change of ownership in the stimulated arm, we compared this correlation with the correlation between perceived valence of touch and change in ownership of the non-stimulated arm as well as with the correlation between perceived valence of touch and change in dissociation using a procedure based on Fisher’s r-to-z transformation. This has been shown to be robust with respect to Type I error, also when applied for non-parametric Spearman correlation [58]. We further used non-parametric partial correlations for testing the relationship between perceived valence of touch and change in arm ownership while controlling for change in state dissociation as assessed by the DSS-4. Since HC experienced constantly high body ownership and constantly low dissociation, both measures lacking substantial variance (see Table S3 in the supplement), we performed these analyses only for the BPD group. As comorbid PTSD has previously been found to influence dissociative experiences [59], we further compared change in dissociation and arm ownership from pre to post stimulation between BPD patients with and without PTSD using Wilcoxon signed-rank tests.

In order to investigate the specificity of alterations in affective somatosensory processing, we compared data of BPD patients and HC for mechanical detection thresholds (MDT), warm perception thresholds (WPT), and heat pain thresholds (HPT) using t-tests for independent samples or Mann-Whitney-U tests. To assess whether there was an association between positive and negative somatosensation, we further correlated perceived valence and intensity of touch with HPT in both groups separately. Due to the small sample sizes for these analyses, the results are reported in the supplement.

Initially, perception of touch in trials with and without startle stimuli was compared in both groups separately using paired t-tests or Wilcoxon signed-rank test. This was done to test for potential effects of the acoustic startle probe on touch perception. Since there were no significant differences in ratings between trials with and without startle probes in either group (see Table S2 in the supplement), we used the mean ratings of touch with and without startle probes for all analyses.

We report test statistics, *p*-values (in case of multiple testing we report Bonferroni-corrected *p*-values, i.e., *p*_Bonf_), and absolute values of effect sizes using Cohen’s *d* (based on pooled *SD*), *r* (applying the equation $=\frac{z}{\sqrt{n}}$), or partial *ƞ*^*2*^. All statistical analyses were performed using IBM SPSS v25.

## Results

### Perception of pleasant touch and affect modulated ASR in BPD and HC participants

**Fig. 2:**
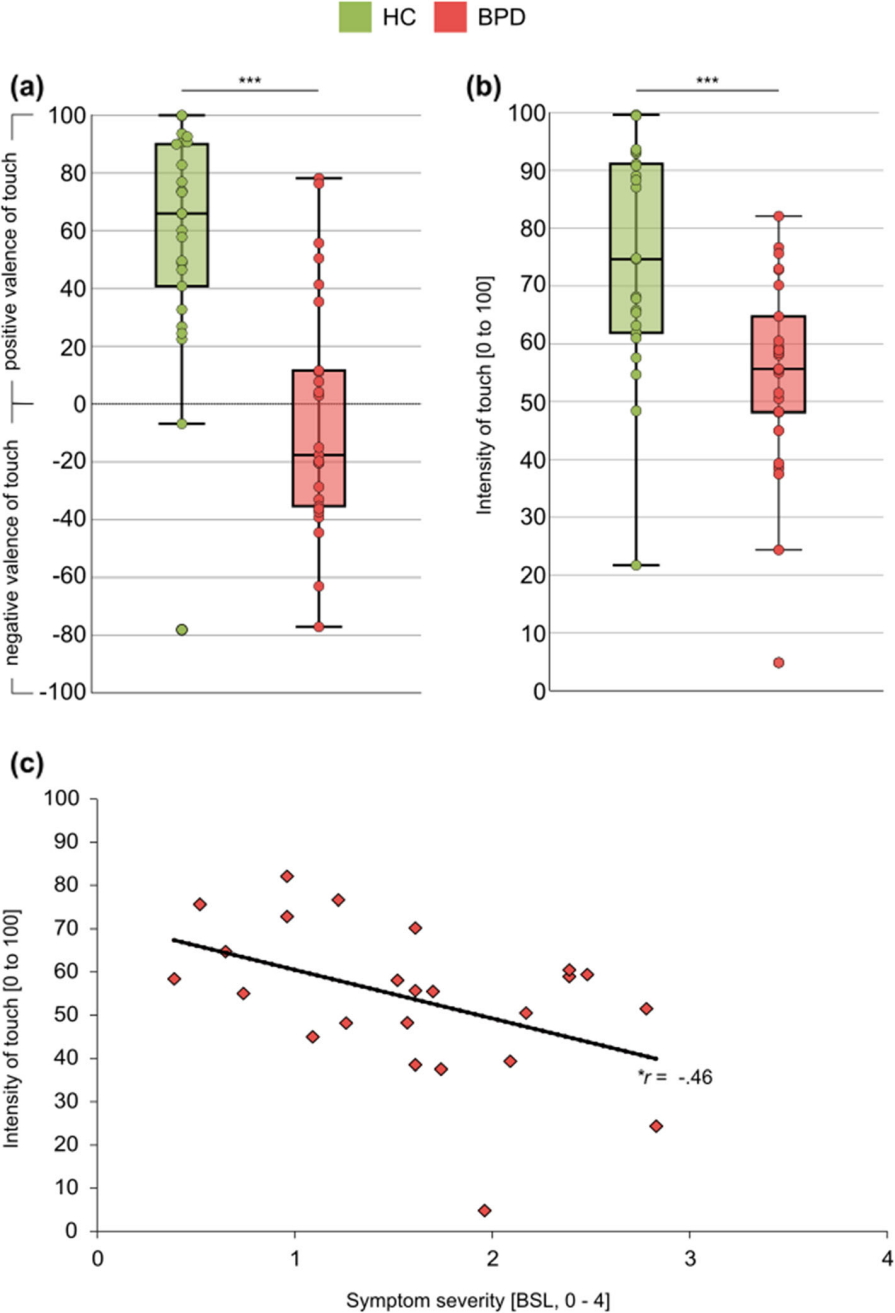
(**a)** Perceived valence of touch in healthy controls (HC) and borderline personality disorder patients (BPD) (**b)** Perceived intensity of touch in HC and BPD. **(c)** Association between perceived intensity of touch and symptom severity as assessed with the Borderline symptom list (BSL) in BPD. Boxplots: Medians and quartiles are marked by the lines of the boxes. Whiskers indicate 1.5 inter-quartile range or minimum/maximum value. Values of single subjects are marked by a dot. * *p* < .05 *** *p* < .001

Perceived valence of touch was significantly lower in participants with BPD (*M* = − 4.50, *SD* = 41.56) compared to HC (*M* = 56.85, *SD* = 39.78), *t* (48) = − 5.33, *p* < .001, *d* = 1.51 (see Fig. 2a). Both groups further differed significantly in the sensory aspect of pleasant touch perception: the intensity ratings were significantly lower in participants with BPD (*Mdn* = 55.67, *IQR* = 20.87) compared to HC (*Mdn* = 74.67, *IQR* = 30.21), *U* = 126.00, *z* = − 3.62, *p* < .001, *r* = .72 (see Fig. 2b). The result of the rank based ANCOVA suggests that, after controlling for the effect of perceived intensity, there was a significant difference in perceived touch valence between both groups, *F* (1,47) = 19.52, *p* < .001, *ƞ*^*2*^ = .29.

In BPD there was a significant negative correlation of symptom severity with perceived intensity of touch (*r* (22) = −.46; *p* = .025) (see Fig. 2c) but not with perceived valence of touch (*r* (22) = .08, *p* = .720).

**Fig. 3:**
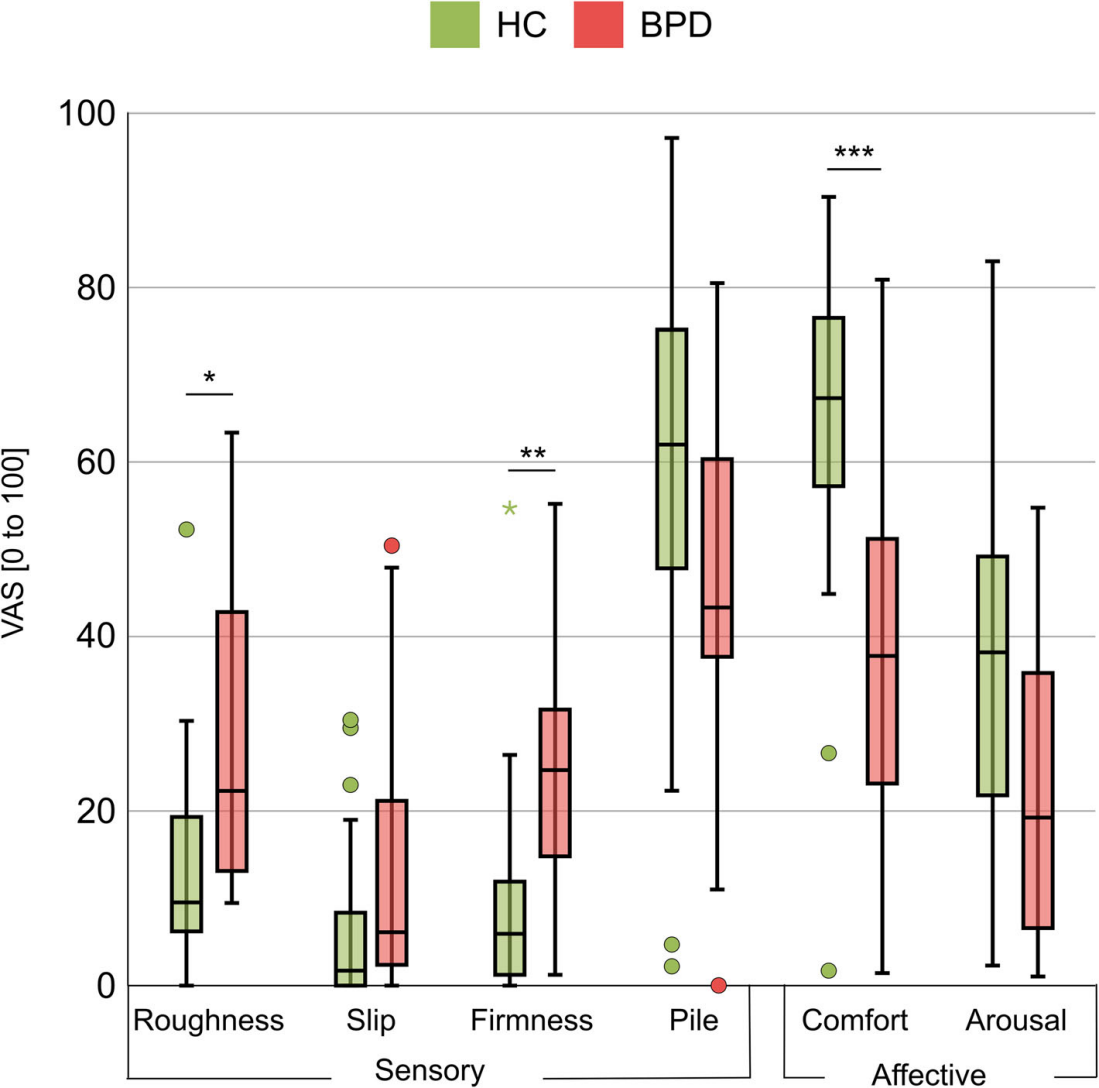
Boxplots for ratings of qualitative aspects of touch perception in healthy controls (HC) and borderline personality disorder patients (BPD). Medians and quartiles are marked by the lines of the boxes. Whiskers indicate 1.5 IQR or minimum/maximum value. Outliers are marked by a dot; extreme values are marked by a colored asterisk. * *p* < .05, ** *p* < .01, *** *p* < .001

Descriptive data for the qualitative aspects of touch for both groups as well as test statistics for the group comparisons can be found in Table 2 and are further visualized in Fig. 3. BPD participants rated the touch as being significantly rougher and firmer compared to the HC group. For the affective component, BPD participants indicated significantly less comfort than the HC group.

**Table 2:** Pleasant touch perception in participants with borderline personality disorder (BPD) and healthy controls (HC)

	BPD (*n* = 25)	HC (*n* = 25)	Statistics
	*M* (*SD*)	*M* (*SD*)	
	*Mdn (IQR)*	*Mdn (IQR)*	
**Valence**	−4.50 (41.56)	56.85 (39.78)	t(48) = −5.33, p < .001
	−17.58 (59.29)	65.83 (53.54)	
**Intensity**	54.58 (17.30)	74.64 (19.15)	U = 126.00, z = −3.62, p < .001
	55.67 (20.87)	74.67 (30.21)	
Sensory components			
**Roughness**	27.80 (16.53)	13.49 (11.33)	U = 128.00, z = −3.58, p_Bonf_ = .002
	22.31 (31.72)	9.50 (13.63)	
**Slip**	14.08 (16.03)	7.22 (9.83)	U = 216.00, z = −1.89, p_Bonf_ = .349
	6.08 (21.83)	1.67 (13.00)	
**Firmness**	23.44 (14.57)	9.15 (11.93)	U = 121.50, z = −3.71, p_Bonf_ = .001
	24.70 (22.00)	5.90 (11.30)	
**Pile**	47.13 (19.83)	57.37 (25.79)	U = 215.00, z = −1.89, p_Bonf_ = .354
	43.33 (24.33)	62.00 (38.42)	
Affective components			
**Comfort**	36.81 (19.88)	64.22 (19.88)	t(48) = −4.87, p_Bonf_ < .001
	37.78 (30.89)	67.33 (22.69)	
**Arousal**	22.72 (16.99)	35.45 (20.19)	t(48) = −2.41, p_Bonf_ = .119
	19.25 (31.25)	38.19 (27.56)	

BPD = borderline personality disorder; HC = healthy control; n = number; *M* = mean; *SD* = standard deviation; *Mdn* = median; *IQR* = interquartile range; p_Bonf_ = Bonferroni corrected *p*-value

There was no significant difference in affect-modulated ASR between BPD (*M* = − 6.22 μV, *SD* = 23.04) and HC (*M* = − 2.55 μV, *SD* = 25.17), *t* (37) = − 0.47, *p* = .638, *d* = 0.15. Affect-modulated ASR was not significantly related to perceived valence of touch in BPD (*r* (17) = −.13, *p* = .589) or HC (*r* (18) = .15, *p* = .538). There was also no significant correlation between affect-modulated ASR and perceived intensity of touch (BPD: *r* (17) = .33, *p* = .162; HC: *r*_*s*_ (18) = .31, *p* = .182).

### Pleasant touch and dissociative states in BPD

**Fig. 4:**
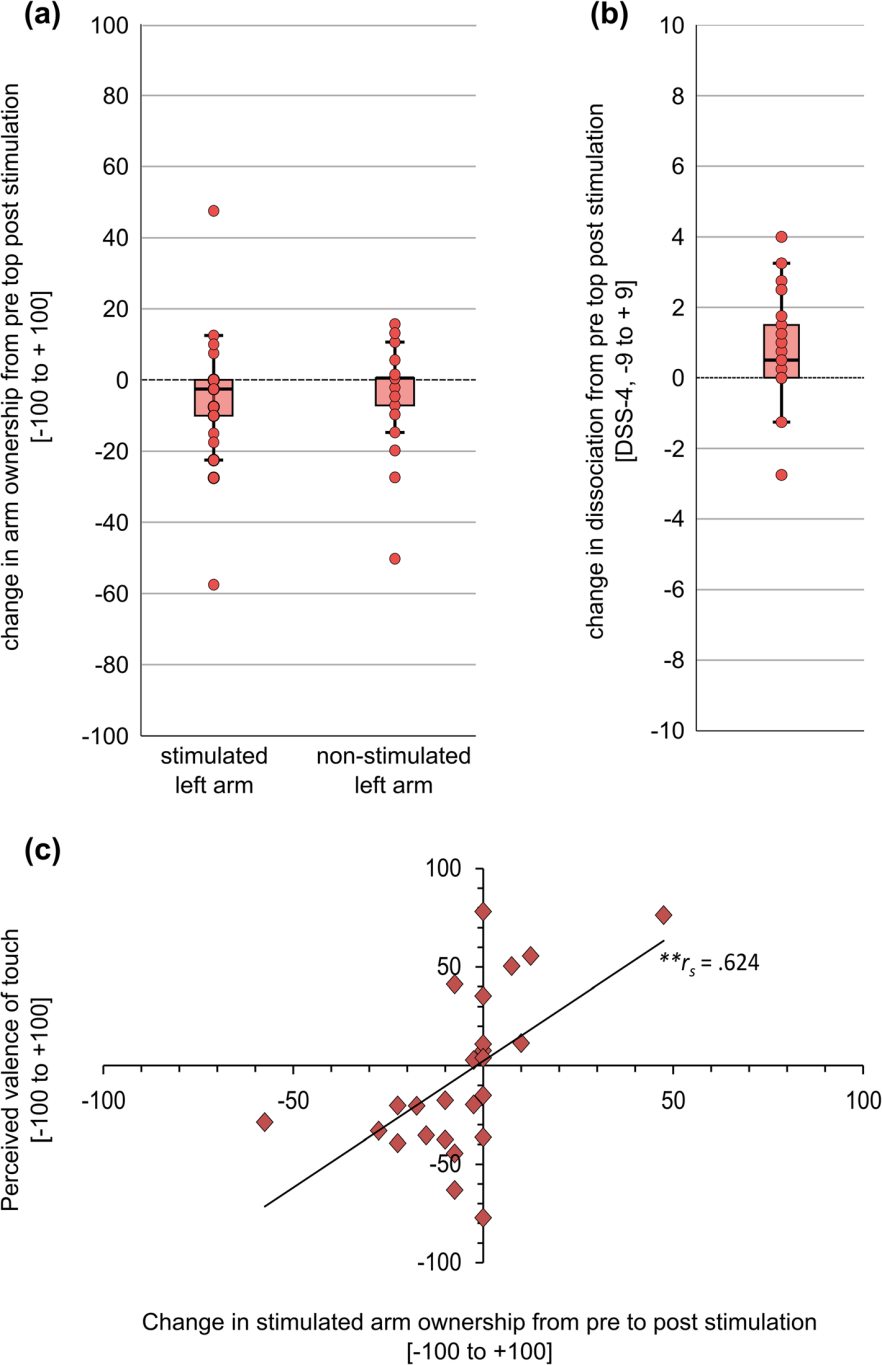
**(a)** Change in body ownership experiences from pre to post stimulation in borderline personality disorder patients (BPD); **(b)** Change in dissociation as assessed with the Dissociation-Tension scale acute (DSS-4) from pre to post stimulation in BPD; **(c)** Association between perceived valence of touch and change in stimulated arm ownership from pre to post pleasant touch perception in borderline personality disorder. ** *p* < .01

Descriptives for state dissociation and body ownership distortions before and after stimulation as well as the respective change scores can be found in Table 3. The changes from pre to post stimulation are further visualized in Fig. 4 a) and b). In BPD, state dissociation prior to the experiment was negatively correlated with perceived intensity of touch (*r* (23) = −.480, *p*_*Bonf*_ = .045) but not with perceived valence of touch (*r* (23) = −.079, *p*_*Bonf*_ > .999). There was no significant association between body ownership experiences prior to the experiment and perceived valence or intensity of touch (all *r*_*s*_ ≤ |.232|, all *p*_*Bonf*_ ≥ .558.)

**Table 3:** Body ownership and state dissociation before and after stimulation with pleasant touch in borderline personality disorder patients

	Pre *M* (*SD*) *Mdn (IQR)*	Post *M* (*SD*) *Mdn (IQR)*	Change *M* (*SD*) *Mdn (IQR)*
**Body ownership stimulated left arm [%]**	74.50 (28.80) 87.50 (46.25)	69.20 (29.56) 67.50 (52.50)	-5.30 (18.09) -2.50 (12.50)
**Body ownership non stimulated right arm [%]**	78.50 (24.58) 87.50 (37.50)	74.44 (26.69) 82.50 (45.00)	-4.06 (13.57) 0.00 (9.25)
**State dissociation (DSS-4)**	1.85 (1.42) 1.50 (2.38)	2.68 (2.24) 2.00 (4.25)	0.83 (1.44) 0.50 (1.63)

Pre = before pleasant touch application; Post = after pleasant touch application, Change = Post-Pre; *M* = mean; *SD* = standard deviation; *Mdn* = median; *IQR* = interquartile range; DSS-4 = Short version of the Dissociation tension scale acute [53]

In BPD, there was a significant increase in state dissociation from pre to post pleasant touch stimulation, *t* (24) = − 2.87, *p*_*Bonf*_ = .024, *d* = 0.88 (see Table 3). However, there was no significant pre-post difference for body ownership, neither for the stimulated left arm, *z* = − 1.80, *p*_*Bonf*_ = .219, *r* = .25, nor the non-stimulated right arm, *z* = − 1.19, *p*_*Bonf*_ = .738, *r* = .17 (see Table 3).

Perceived valence of touch was significantly positively correlated with changes in ownership of the stimulated left arm in BPD (*r*_*s*_ (23) = .624, *p*_*Bonf*_ = .003, see Fig. 4c). Results of a bootstrapping procedure (10,000 samples) revealed a BCa (Bias corrected and accelerated) 95% CI [.306, .821], indicating that it is a robust correlation. There was no significant correlation between perceived valence of touch and change in ownership of the non-stimulated right arm (*r*_*s*_ (23) = .221, *p*_*Bonf*_ = .867) and change in state dissociation (*r*_*s*_ (23) = −.262 *p*_*Bonf*_ = .618) in BPD. The correlation between perceived valence of touch and change of ownership in the stimulated arm significantly differed from the correlation between perceived valence and change of ownership in the non-stimulated arm (*z* = 2.43, *p*_*Bonf*_ = .030) as well as from the correlation between perceived valence and change of dissociation (*z* = 2.68, *p*_*Bonf*_ = .014), indicating that the association between perceived valence of touch and change in ownership was specific for the stimulated arm. However, change in state dissociation was significantly related to both change in ownership of the stimulated left arm (*r*_s_ (23) = −.588, *p*_*Bonf*_ = .004) and the non-stimulated arm (*r*_*s*_ (23) = −.556, *p*_*Bonf*_ = .004). After controlling for the change in state dissociation, perceived valence of touch was still significantly related to the change in ownership of the stimulated left (*r*_*s*_ (22) = .603, *p*_*Bonf*_ = .004), but not the right non-stimulated arm (*r*_*s*_ (22) = .094, *p*_*Bonf*_ > .999). There was no significant correlation between the perceived intensity of touch and change in dissociative levels (all *r*_*s*_ ≤ |.288|. all *p*_*Bonf*_ ≥ .489).

There was no significant difference in the change in ownership from pre to post stimulation between BPD with and without PTSD for the stimulated arm (*U* = 29.50, *z* = − 1.42, *p* = .156) or the non-stimulated arm (*U* = 50.00, *z* = 0.00*, p* > .999). There was also no significant difference for changes in general dissociation as assessed with the DSS-4 when comparing these subgroups of BPD patients (*U* = 27.50, *z* = − 1.56, *p* = .120).

## Discussion

The aim of the present study was to investigate whether pleasant touch perception is disturbed in BPD. We applied standardized pleasant touch stimuli to the back of the left hand of female BPD patients and HC. We assessed the perception of touch as well as its affective processing in terms of affect-modulated acoustic startle responses. In order to explore a potential modulating effect of pleasant touch on state psychopathology, we assessed changes in state dissociation and dissociative body experiences in terms of reduced body ownership, before and after touch application. We further investigated the specificity of somatosensory alterations and aimed to test whether the perception of somatosensory stimuli with positive and negative valence is interrelated.

As expected, the perceived valence of pleasant touch was less positive in BPD compared to HC. After controlling for the potentially confounding effect of perceived intensity of touch, perceived valence of touch still remained less positive in individuals with BPD compared to HC with an effect of *ƞ*^*2*^ = .29. We further found alterations in qualitative aspects of touch perception in BPD compared to HC, which (a) confirmed our main results regarding reduced positive perception of pleasant touch in terms of reduced comfort ratings in BPD compared to HC and (b) revealed differences in certain sensory aspects of touch. Thus, BPD patients rated the perceived touch rougher and firmer compared to HC. Finally, our results suggest that perceived valence of touch was related to changes in dissociative body perception in terms of disturbed body ownership, especially of the stimulated body site, but not to the dissociative state in general. The positive correlation between perceived valence of touch and the change in arm ownership of the stimulated site suggests that a more unpleasant perception of touch is associated with a decrease in body ownership experiences from pre to post stimulation, while a more positive perception of touch was related with an increase in reported arm ownership.

Our main result of pleasant touch disturbances in BPD is in line with previous results indicating that perception of positively valenced stimuli is altered in the disorder [5–8]. It extends previous findings on somatosensory alterations in BPD by suggesting that not only pain perception, but also the perception of its positive counterpart is altered in BPD. Together, this supports the assumption of altered affective somatosensory processing in BPD. Interestingly, similar to our results on altered qualitative touch experiences, a recent study found alterations in qualitative pain ratings in BPD, in terms of a specific loss of the pain component sharpness [60]. Qualitatively changed somatosensory perception might be due to altered evaluation of negative [60] and positive somatosensory stimuli and related cognitive processes. There is a common neurobiology of pain and pleasure [23] and evidence for cognitive top-down modulation of both. It has been shown that top-down cognitive factors can influence the affective representation of touch in healthy subjects [61]. For the pain domain, cognitive down-regulation of the affective pain component has been proposed as an antinociceptive mechanism in BPD, represented by an interaction between prefrontal and limbic areas [16]. Taken together, it can be assumed that a dysregulation of cortico-limbic pathways might be an underlying neural mechanism for altered affective somatosensation in BPD.

Unexpectedly, we found not only alterations in the affective component of pleasant touch but also a reduced touch sensitivity, in terms of heightened mechanical detection threshold and reduced perceived intensity of pleasant touch. This suggests that for the touch domain both, perception of the affective and sensory component might be altered. Interestingly, only perceived intensity of touch but not perceived valence of touch, was associated with symptom severity and state dissociation in BPD. Similarly, Bekrater-Bodmann et al. [9] reported an association between state dissociation and the magnitude of the pain percept but not with affective pain perception in terms of perceived unpleasantness. Altered gating and reduced processing of sensory input in acute dissociation [62] might mainly affect perceived intensity of a somatosensory stimulus. In consequence, higher levels of dissociation in BPD might decrease the salience of somatosensory stimuli, making it more difficult for them to be perceived, as has been proposed before [18]. This might be true especially for the sensory aspects of pain and (pleasant) touch, stimuli with a relatively high salience. For exteroception as assessed by two point discrimination or proprioception as assessed by weight-discrimination there is no evidence of altered perception in BPD [17]. However, the alteration of the affective component of pleasant touch was independent of reduced touch sensitivity, indicating that at least partly distinct processes might underlie alterations in the sensory and affective components of touch processing.

The missing association between perceived valence of touch and symptom severity in BPD raises the question whether alterations in affective touch perception are disorder-specific or reflect unspecific alterations in psychopathological states. Studies on other mental disorders are sparse, but there is first evidence of altered pleasant touch processing in current [63, 64] and remitted [65] anorexia nervosa, as well as posttraumatic stress disorder [66], all of which are common comorbidities in BPD [67]. The assumption of an unspecific alteration might further be supported by the results of Croy et al. [24] who reported reduced pleasant perception of touch in a heterogeneous sample of psychiatric outpatients suffering from different mental disorders. Interestingly, a recent study found that pleasant touch perception is affected by disorganized attachment [68], an attachment style characterized by inconsistent attachment behavior, which is overrepresented in personality disorders such as BPD [69] as well as patients suffering from anorexia nervosa [70], and which has been linked to psychological traumatization [71]. According to attatchment research, disorganized attachment is often a second-generation effect characterized by frightening and/or frightened parental interaction by caregivers suffering themselves from attachment-related trauma or losses [72, 73]. Touch perception is the earliest sensory modality to develop [74] and might thus be particularly prone to adapting to adverse developmental circumstances [75]. Thus, from an etiological perspective, it can be speculated that growing up in a frightening environment, where caregivers do not represent a secure base, might result in disturbed interpretation of safety signals like pleasant touch, which, depending on other contributing factors (e.g. certain genotypes or other environmental factors), could manifest in various psychopathological states.

From a psychopathologial perspective, the results of our study have some important implications. Mainly, our data suggest that more negative perception of touch is associated with a further decrease in body ownership experiences in BPD, while on the other hand touch stimulation, which is perceived as pleasant, might have beneficial effects on disturbed body ownership experiences of the stimulated limb in BPD. Thus our results point out that the effect of stimulation on body ownership experiences in BPD depends not only on the properties of the touching stimulus but its perceived valence might also play an important role. This differs from the results of Jenkinson et al. [35], who found an increase in body ownership experiences after pleasant touch stimulation of the affected limb of stroke patients, which was not associated with perceived valence. The authors propose that increased body ownership experiences might be the result of integrating new sensation from the affected part with one’s multimodal self-representation. However, they only report positive touch perception, which might foster an embodied self. A negative perception of touch, as present in some of the BPD subjects of this study, might hinder or even reverse this integrative process. Results of a recent study suggest that uncertainty and affective incongruences can disrupt the multisensory integration process that leads to the experience of body ownership [76], highlighting the importance of top-down processes, for example, information processing guided by higher-level knowledge and expectations, for the experience of body ownership. Expectations or anticipation of the affective input were not assessed in the current study. However, based on a spontaneous statement of one BPD subject after the present experiment who stated that the applied touch “felt like the touch of someone who wants to comfort you but doesn’t mean it”, it is conceivable that there might be a high level of perceived inconsistency for some BPD patients during touch experiences. Thereby, negative self-evaluations, which are common in BPD [77, 78], might play an important role, as they have been suggested to result in a devaluation of self-referential positive experiences [79]. To further assess hypothesized top-down influences on the effect of pleasant touch stimulation on body ownership experiences, future studies might combine pleasant touch stimulation with other affective stimuli, for example, affective pictures or other self-relevant stimuli.

Moreover, reduction of body ownership experiences from pre to post stimulation might also be the result of a coping process. As proposed by trauma models of dissociation, dissociative responses, including the experience of being detached from one’s own body, may be a mechanism to cope with overwhelming experiences especially in threatening situations without chance to escape [e.g. 73]. Further, it has been proposed that trauma-related memories and re-experiencing symptoms might be specifically triggered by perceptual stimuli associated with the traumatic event [80]. Even though there was no evidence for differences in BPD with and without PTSD in the current study, it is possible that for some BPD patients with specific traumatic experiences the pleasant touch stimulation during the experiment might have reactivated traumatic experiences when touch was associated with negative experiences [81], which in turn might result in unpleasant touch perception and related reduction in body ownership experience. To probe this hypothesis, further studies with larger sample sizes comparing BPD individuals with and without traumatic experiences might take into account the type of trauma and its association with perception of pleasant touch stimuli as well as body-related psychopathology.

From a more clinical perspective, our results suggest that touch stimulation, which is perceived as pleasant, might be a promising candidate to target reduced body ownership experiences in BPD, which have been shown to normalize in the remitted state of the disorder [37]. Therefore, it might be important to create a situation where the patient feels safe and anticipates a positive incoming signal. Individualized positive cues or being touched with materials that are positively connotated might help to re-evaluate incoming pleasant touch-signals.

### Limitations and outlook

Several limitations of our study must be noted. First, sample sizes, especially for the assessment of thermal perception and pain thresholds, were relatively small. Even though our supplemental results indicate an association between perception of positive and negative somatosensation in HC, there was no significant correlation between altered heat pain threshold and pleasant touch perception in BPD. However, interpretation of this result is limited not only due to the small sample size but also because pain thresholds reflect only one facet of altered pain perception in BPD. In future studies, beyond thresholds, the assessment of sensory, affective, and qualitative aspects of positive and negative somatosensory stimuli might be necessary to elucidate somatosensory alteration in BPD. Another limitation is that the intake of SSRIs was not interrupted in this study. SSRIs have been successfully used to treat chronic pain [82] and sensory alterations as possible side effects cannot be ruled out. All subjects in the present study were female, limiting the generalizability of our results. Previous results on a gender effect of pleasant touch perception are mixed, with some studies indicating that female subjects perceive touch as more pleasant [83, 84], but there is also a study indicating that there is no significant gender effect [85]. Furthermore, there was no control condition where touch was applied with a velocity outside the range of CT optimal velocities [e.g. 55,60] and we did not test if differences in perceived valence and intensity of touch between BPD and HC might be extended to touch applied with non-CT velocities. Therefore, future studies are necessary to investigate whether alterations in touch perception in BPD relate specifically to the CT system. We further did not include a clinical control sample to investigate disorder-specific effects. Future studies on pleasant touch perception in BPD might further include samples suffering from eating disorder and PTSD, as both are common comorbidities in BPD and have been shown to be related to disturbances in pleasant touch perception [63–66]. Moreover, expanding the investigation of patients by incorporating dimensional approaches might be helpful to disentangle the mechanisms behind disturbances in pleasant touch processing in BPD and its relationship with dissociative experiences. Finally, even if the difference in perceived valence of touch was obvious on a perceptual level, we could not provide evidence for differences between both groups in its physiological correlate, in terms of affect-modulated ASR. Affect-modulated ASR was not previously tested in pleasant touch studies in HCs before and the missing association between affect-modulated ASR and perceived valence of touch in HC raises the question whether ASR is an appropriate peripheral physiological correlate for the specific case of pleasant touch perception at all. EMG of the of the zygomaticus major (smile) muscle might be a more suitable physiological correlate of pleasant touch perception [86].

Future studies also need to investigate the association between altered touch perception and deficits in social interaction. Pleasant touch plays an important role for initiating affiliative interaction, the maintenance of social bonds, contributes to the nonverbal communication of emotions [87] and reduces feelings of social exclusion [88], all social functions that are impaired in BPD [89].

## Conclusion

The results of the current study provide novel empirical findings that pleasant touch perception is altered in BPD. A complex and partly distinct mechanism might underlie alterations in sensory and affective aspects of somatosensation, and accordingly, disturbances in sensory and affective processing might be differentially related to BPD psychopathology. Altered evaluation of pleasant touch might be related to negative self-evaluation and traumatic experiences and could play an important role in impairments in social interaction, as pleasant touch is a basic affiliative social signal. A deeper understanding of the mechanisms behind altered processing of pleasant touch and the effects of pleasant touch stimulation might help in the development of innovative treatment approaches, as our results indicate that there might be beneficial effect of pleasant touch stimulation on state psychopathologies in case of positive touch perception. If future studies reveal antecedents of positive touch perception in BPD, a positively valenced somatosensory stimulation might serve as a substitute action for self-infliction of pain in terms of nonsuicidal self-injury behavior which is common in BPD [90] and primarily motivated by a reduction of aversive inner tension and related dissociative states [91].

## Supplementary Information

**Additional file 1.** Development of a German set of qualitative descriptors for pleasant touch perception.

## Data Availability

The data supporting the findings of this study are available from the corresponding author upon reasonable request.

## Abbreviations

ASR: Acoustic startle response
BDI: Beck Depression Inventory
BPD: Borderline personality disorder
BSL-23: Borderline symptom list (short version)
CT: C-tactile
DSM: Diagnostic and statistical manual for mental disorders
DSS-4: Dissociation-tension scale acute (short version)
EMG: Electromyography
HC: Healthy controls
HPT: Heat pain thresholds
IPDE: International personality disorder examination
MDT: Mechanical detection threshold
PT: Pleasant touch
QST: Quantitative sensory testing
SSRIs: Selective serotonin reuptake inhibitors
STAI: State-trait-anxiety inventory
TPT: Touch perception task
WPT: Warm perception threshold
VAS: Visual analogue scale

## References

[1] Lieb K, Zanarini MC, Schmahl C, Linehan PMM, Bohus PM (2004). Borderline personality disorder. Lancet.

[2] Carpenter RW, Trull TJ (2013). Components of emotion dysregulation in borderline personality disorder: a review. Curr Psychiatr Rep.

[3] Schulze L, Schmahl C, Niedtfeld I (2016). Neural correlates of disturbed emotion processing in borderline personality disorder: a multimodal Meta-analysis. Biol Psychiatr.

[4] Bertsch K, Hillmann K, Herpertz SC (2018). Behavioral and neurobiological correlates of disturbed emotion processing in borderline personality disorder. Psychopathology.

[5] Fenske S, Lis S, Liebke L, Niedtfeld I, Kirsch P, Mier D (2015). Emotion recognition in borderline personality disorder: effects of emotional information on negative bias. Borderline Personal Disord Emot Dysregulation.

[6] Thome J, Liebke L, Bungert M, Schmahl C, Domes G, Bohus M, Lis S (2016). Confidence in facial emotion recognition in borderline personality disorder. Personal Disord Theory Res Treat.

[7] Arrondo G, Murray GK, Hill E, Szalma B, Yathiraj K, Denman C, Dudas RB (2015). Hedonic and disgust taste perception in borderline personality disorder and depression. Br J Psychiatry.

[8] Reichenberger J, Eibl JJ, Pfaltz M, Wilhelm FH, Voderholzer U, Hillert A, Blechert J (2017). Don’t praise me, don’t chase me: emotional reactivity to positive and negative social-evaluative videos in patients with borderline personality disorder. J Personal Disord.

[9] Bekrater-Bodmann R, Chung BY, Richter I, Wicking M, Foell J, Mancke F, Schmahl C, Flor H (2015). Deficits in pain perception in borderline personality disorder: results from the thermal grill illusion. Pain.

[10] Chung BY, Hensel S, Schmidinger I, Bekrater-Bodmann R, Flor H (2020). Dissociation proneness and pain hyposensitivity in current and remitted borderline personality disorder. Eur J Pain.

[11] Ludäscher P, Bohus M, Lieb K, Philipsen A, Jochims A, Schmahl C (2007). Elevated pain thresholds correlate with dissociation and aversive arousal in patients with borderline personality disorder. Psychiatry Res.

[12] Ludäscher P, Greffrath W, Schmahl C, Kleindienst N, Kraus A, Baumgärtner U, Magerl W, Treede RD, Bohus M (2009). A cross-sectional investigation of discontinuation of self-injury and normalizing pain perception in patients with borderline personality disorder. Acta Psychiatr Scand.

[13] Russ MJ, Roth SD, Lerman A, Kakuma T, Harrison K, Shindledecker RD, Hull J, Mattis S (1992). Pain perception in self-injurious patients with borderline personality disorder. Biol Psychiatry.

[14] Schmahl C, Greffrath W, Baumgärtner U, Schlereth T, Magerl W, Philipsen A, Lieb K, Bohus M, Treede RD (2004). Differential nociceptive deficits in patients with borderline personality disorder and self-injurious behavior: laser-evoked potentials, spatial discrimination of noxious stimuli, and pain ratings. Pain.

[15] Schmahl C, Meinzer M, Zeuch A, Fichter M, Cebulla M, Kleindienst N, Ludäscher P, Steil R, Bohus M (2010). Pain sensitivity is reduced in borderline personality disorder, but not in posttraumatic stress disorder and bulimia nervosa. World J Biol Psychiatr.

[16] Schmahl C, Bohus M, Esposito F, Treede RD, Di Salle F, Greffrath W (2006). Neural correlates of antinociception in borderline personality disorder. Arch Gen Psychiatr.

[17] Pavony MT, Lenzenweger MF (2014). Somatosensory processing and borderline personality disorder: pain perception and a signal detection analysis of proprioception and exteroceptive sensitivity. Personal Disord Theory Res Treat.

[18] Defrin R, Cohen Sagy N, Biran I, Goor-Aryeh I, Shai R, Ginzburg K (2020). Enhanced pain modulation capacity among individuals with borderline personality disorder: a possible mechanism underlying their hypoalgesia. Eur J Pain.

[19] Taneja P, Olausson H, Trulsson M, Svensson P, Baad-Hansen L (2021). Defining pleasant touch stimuli: a systematic review and meta-analysis. Psychol Res.

[20] Löken LS, Wessberg J, Morrison I, McGlone F, Olausson H (2009). Coding of pleasant touch by unmyelinated afferents in humans. Nat Neurosci.

[21] Olausson H, Lamarre Y, Backlund H, Morin C, Wallin BG, Starck G, Ekholm S, Strigo I, Worsley K, Vallbo ÅB, Bushnell MC (2002). Unmyelinated tactile afferents signal touch and project to insular cortex. Nat Neurosci.

[22] McGlone F, Olausson H, Boyle JA, Jones-Gotman M, Dancer C, Guest S, Essick G (2012). Touching and feeling: differences in pleasant touch processing between glabrous and hairy skin in humans. Eur J Neurosci.

[23] Leknes S, Tracey I (2008). A common neurobiology for pain and pleasure. Nat Rev Neurosci.

[24] Croy I, Geide H, Paulus M, Weidner K, Olausson H (2016). Affective touch awareness in mental health and disease relates to autistic traits – an explorative neurophysiological investigation. Psychiatry Res.

[25] Lang PJ, Bradley MM, Cuthbert BN (1990). Emotion, attention, and the startle reflex. Psychol Rev.

[26] Kuhn M, Wendt J, Sjouwerman R, Büchel C, Hamm A, Lonsdorf TB (2020). The Neurofunctional basis of affective startle modulation in humans: evidence from combined facial electromyography and functional magnetic resonance imaging. Biol Psychiatr.

[27] Hurlemann R, Arndt S, Schlaepfer TE, Reul J, Maier W, Scheele D (2015). Diminished appetitive startle modulation following targeted inhibition of prefrontal cortex. Scand J Psychol.

[28] Krause-Utz A, Winter D, Niedtfeld I, Schmahl C (2014). The latest neuroimaging findings in borderline personality disorder topical collection on personality disorders. Curr Psychiatr Rep.

[29] Hazlett EA, Speiser LJ, Goodman M, Roy M, Carrizal M, Wynn JK, Williams WC, Romero M, Minzenberg MJ, Siever LJ, New AS (2007). Exaggerated affect-modulated startle during unpleasant stimuli in borderline personality disorder. Biol Psychiatr.

[30] Limberg A, Barnow S, Freyberger HJ, Hamm AO (2011). Emotional vulnerability in borderline personality disorder is cue specific and modulated by traumatization. Biol Psychiatr.

[31] Herpertz SC, Kunert HJ, Schwenger UB, Sass H (1999). Affective responsiveness in borderline personality disorder: a psychophysiological approach. Am J Psychiatry.

[32] Crucianelli L, Metcalf NK, Fotopoulou AK, Jenkinson PM (2013). Bodily pleasure matters: velocity of touch modulates body ownership during the rubber hand illusion. Front Psychol.

[33] Panagiotopoulou E, Filippetti ML, Tsakiris M, Fotopoulou A (2017). Affective touch enhances self-face recognition during multisensory integration. Sci Rep.

[34] Longo MR, Schüür F, Kammers MPM, Tsakiris M, Haggard P (2008). What is embodiment? A psychometric approach. Cognition.

[35] Jenkinson PM, Papadaki C, Besharati S, Moro V, Gobbetto V, Crucianelli L, et al. Welcoming back my arm: affective touch increases body ownership following right-hemisphere stroke. Brain Commun. 2020;2(1). 10.1093/BRAINCOMMS/FCAA034. 10.1093/braincomms/fcaa034 PMC7425337 32954292

[36] American Psychiatric Association (2013). Diagnostic and statistical manual of mental disorders (DSM-5®).

[37] Löffler A, Kleindienst N, Cackowski S, Schmidinger I, Bekrater-Bodmann R (2020). Reductions in whole-body ownership in borderline personality disorder–a phenomenological manifestation of dissociation. J Trauma Dissociation.

[38] Schmahl C, Herpertz SC, Bertsch K, Ende G, Flor H, Kirsch P, Lis S, Meyer-Lindenberg A, Rietschel M, Schneider M, Spanagel R, Treede RD, Bohus M (2014). Mechanisms of disturbed emotion processing and social interaction in borderline personality disorder: state of knowledge and research agenda of the German clinical research unit. Borderline Personal Disord Emot Dysregulation.

[39] Oldfield RC (1971). The assessment and analysis of handedness: the Edinburgh inventory. Neuropsychologia.

[40] Fava GA, Gatti A, Belaise C, Guidi J, Offidani E (2015). Withdrawal symptoms after selective serotonin reuptake inhibitor discontinuation: a systematic review. Psychother Psychosom.

[41] American Psychiatric Association (2000). Diagnostic criteria from DSM-IV-TR.

[42] Wittchen H-U, Zaudig M, Fydrich T (1997). SKID. Strukturiertes Klinisches Interview für DSM-IV. Achse I und II.

[43] Loranger AW, Janca A, Norman S (1997). Assessment and diagnosis of personality disorders: the ICD-10 international personality disorder examination (IPDE).

[44] Bohus M, Kleindienst N, Limberger MF, Stieglitz R-D, Domsalla M, Chapman AL, Steil R, Philipsen A, Wolf M (2009). The short version of the borderline symptom list (BSL-23): development and initial data on psychometric properties. Psychopathology.

[45] Hautzinger M, Bailer M, Worall H, Keller F (1995). Beck-depression-Inventar (BDI).

[46] Laux L, Glanzmann P, Schaffner P, Spielberger CD (1981). Das State-Trait-Angstinventar (STAI-G). Theoretische Grundlagen und Handanweisungen.

[47] Nees F, Usai K, Löffler M, Flor H (2019). The evaluation and brain representation of pleasant touch in chronic and subacute back pain. Neurobiol Pain.

[48] Cacioppo JT, Berntson GG (1994). Relationship between attitudes and evaluative space: a critical review, with emphasis on the Separability of positive and negative substrates. Psychol Bull.

[49] Norris CJ, Gollan J, Berntson GG, Cacioppo JT (2010). The current status of research on the structure of evaluative space. Biol Psychol.

[50] Watson D, Tellegen A (1985). Toward a consensual structure of mood. Psychol Bull.

[51] Guest S, Dessirier JM, Mehrabyan A, McGlone F, Essick G, Gescheider G, Fontana A, Xiong R, Ackerley R, Blot K (2011). The development and validation of sensory and emotional scales of touch perception. Atten Percept Psychophys.

[52] Stiglmayr C, Schmahl C, Bremner JD, Bohus M, Ebner-Priemer U (2009). Development and psychometric characteristics of the DSS-4 as a short instrument to assess dissociative experience during neuropsychological experiments. Psychopathology.

[53] Blumenthal TD, Cuthbert BN, Filion DL, Hackley S, Lipp OV, Van Boxtel A (2005). Committee report: guidelines for human startle eyeblink electromyographic studies. Psychophysiology.

[54] Lissek S, Biggs AL, Rabin SJ, Cornwell BR, Alvarez RP, Pine DS, Grillon C (2008). Generalization of conditioned fear-potentiated startle in humans: experimental validation and clinical relevance. Behav Res Ther.

[55] Rolke R, Baron R, Maier C, Tölle TR, Treede RD, Beyer A (2006). Quantitative sensory testing in the German research network on neuropathic pain (DFNS): standardized protocol and reference values. Pain.

[56] Leung AY, Wallace MS, Schulteis G, Yaksh TL (2005). Qualitative and quantitative characterization of the thermal grill. Pain.

[57] Conover WJ, Iman RL (1982). Analysis of covariance using the rank transformation. Biometrics.

[58] Myers L, Sirois MJ. Spearman correlation coefficients. Differ Between Encycl Stat Sci. 2006. 10.1002/0471667196.ESS5050.PUB2.

[59] Vermetten E, Spiegel D (2014). Trauma and Dissociation: Implications for Borderline Personality Disorder. Curr Psychiatr Rep.

[60] Schloss N, Shabes P, Kuniss S, Willis F, Treede R, Schmahl C (2019). Differential perception of sharp pain in patients with borderline personality disorder. Eur J Pain.

[61] McCabe C, Rolls ET, Bilderbeck A, McGlone F (2008). Cognitive influences on the affective representation of touch and the sight of touch in the human brain. Soc Cogn Affect Neurosci.

[62] Krause-Utz A, Elzinga B (2018). Current understanding of the neural mechanisms of dissociation in borderline personality disorder. Curr Behav Neurosci Rep.

[63] Crucianelli L, Cardi V, Treasure J, Jenkinson PM, Fotopoulou A (2016). The perception of affective touch in anorexia nervosa. Psychiatry Res.

[64] Davidovic M, Karjalainen L, Starck G, Wentz E, Björnsdotter M, Olausson H (2018). Abnormal brain processing of gentle touch in anorexia nervosa. Psychiatr Res Neuroimaging.

[65] Crucianelli L, Demartini B, Goeta D, Nisticò V, Saramandi A, Bertelli S, Todisco P, Gambini O, Fotopoulou A (2020). The anticipation and perception of affective touch in women with and recovered from anorexia nervosa. Neuroscience.

[66] Strauss T, Rottstädt F, Sailer U, Schellong J, Hamilton JP, Raue C, Weidner K, Croy I (2019). Touch aversion in patients with interpersonal traumatization. Depress Anxiety.

[67] Zimmerman M, Mattia JI (1999). Axis I diagnostic comorbidity and borderline personality disorder. Compr Psychiatry.

[68] Spitoni GF, Zingaretti P, Giovanardi G, Antonucci G, Galati G, Lingiardi V, Cruciani G, Titone G, Boccia M (2020). Disorganized attachment pattern affects the perception of affective touch. Sci Rep.

[69] Westen D, Nakash O, Thomas C, Bradley R (2006). Clinical assessment of attachment patterns and personality disorder in adolescents and adults. J Consult Clin Psychol.

[70] Delvecchio E, Di Riso D, Salcuni S, Lis A, George C (2014). Anorexia and attachment: dysregulated defense and pathological mourning. Front Psychol.

[71] Liotti G (2004). Trauma, dissociation, and disorganized attachment: three strands of a single braid. Psychother Theory Res Pract Train.

[72] Main M, Solomon J, Greenberg MT, Cicchetti D, Cummings EM (1990). Procedures for identifying infants as diorganized/dioriented during the Ainworth Strange Situation. Attach. Presch. years Theory, Res. Interv.

[73] Hesse E, Main M (1999). Psychoanalytic inquiry second-generation effects of unresolved trauma in nonmaltreating parents: dissociated, frightened, and threatening parental behavior. Psychoanal Inq.

[74] Bremner AJ, Spence C (2017). The development of tactile perception. Adv Child Dev Behav.

[75] Crucianelli L, Filippetti ML (2020). Developmental perspectives on interpersonal affective touch. Topoi.

[76] Filippetti ML, Kirsch LP, Crucianelli L, Fotopoulou A (2019). Affective certainty and congruency of touch modulate the experience of the rubber hand illusion. Sci Rep.

[77] Winter D, Bohus M, Lis S (2017). Understanding negative self-evaluations in borderline personality disorder-a review of self-related cognitions, emotions, and motives. Curr Psychiatr Rep.

[78] Kleindienst N, Löffler A, Herzig M, Bertsch K, Bekrater-Bodmann R (2020). Evaluation of the own body in women with current and remitted borderline personality disorder: evidence for long-lasting effects of childhood sexual abuse. Eur J Psychotraumatol.

[79] Winter D, Herbert C, Koplin K, Schmahl C, Bohus M, Lis S (2015). Negative evaluation Bias for positive self-referential information in borderline personality disorder. PLoS One.

[80] Ehlers A, Clark DM. A cognitive model of posttraumatic stress disorder. Behav Res Ther 2000;38:319–345. 10.1016/S0005-7967(99)00123-0, 4. 10.1016/s0005-7967(99)00123-0 10761279

[81] Maier A, Gieling C, Heinen-Ludwig L, Stefan V, Schultz J, Güntürkün O, Becker B, Hurlemann R, Scheele D (2020). Association of Childhood Maltreatment with Interpersonal Distance and Social Touch Preferences in adulthood. Am J Psychiatr.

[82] Patetsos E, Horjales-Araujo E (2016). Treating chronic pain with SSRIs: what do we know?. Pain Res Manag.

[83] Croy I, Angelo SD, Olausson H (2014). Reduced Pleasant Touch Appraisal in the Presence of a Disgusting Odor. PLoS One.

[84] Jönsson EH, Bendas J, Weidner K, Wessberg J, Olausson H, Wasling HB, Croy I (2017). The relation between human hair follicle density and touch perception. Sci Rep.

[85] Sehlstedt I, Ignell H, Wasling HB, Ackerley R, Olausson H, Croy I (2016). Gentle touch perception across the lifespan. Psychol Aging.

[86] Pawling R, Cannon PR, Mcglone FP, Walker SC. C-tactile afferent stimulating touch carries a positive affective value. 2017;12(3). 10.1371/journal.pone.0173457. 10.1371/journal.pone.0173457 PMC5345811 28282451

[87] Björnsdotter M, Morrison · I, Olausson · H. Feeling good: on the role of C Wber mediated touch in interoception. Exp Brain Res 2010;207:149–155. 10.1007/s00221-010-2408-y, 3-4. 10.1007/s00221-010-2408-y 20963582

[88] Von Mohr M, Kirsch LP, Fotopoulou A (2017). The soothing function of touch: affective touch reduces feelings of social exclusion. Sci Rep.

[89] Lis S, Bohus M (2013). Social interaction in borderline personality disorder. Curr Psychiatr Rep.

[90] Zanarini MC, Frankenburg FR, Jager-Hyman S, Reich DB, Fitzmaurice G (2008). The course of dissociation for patients with borderline personality disorder and axis II comparison subjects: a 10-year follow-up study. Acta Psychiatr Scand.

[91] Kleindienst N, Bohus M, Ludäscher P, Limberger MF, Kuenkele K, Ebner-Priemer UW, Chapman AL, Reicherzer M, Stieglitz RD, Schmahl C (2008). Motives for nonsuicidal self-injury among women with borderline personality disorder. J Nerv Ment Dis.

