# Supplementary material for: Pleasant touch perception in borderline personality disorder and its relationship with disturbed body representation
Source: Borderline Personal Disord Emot Dysregul. 2022 Feb 1;9:3. doi: 10.1186/s40479-021-00176-4 (PMC8805331; doi:10.1186/s40479-021-00176-4)
Supplement: Supplementary file 1 — Additional file 1. Development of a German set of qualitative descriptors for pleasant touch perception. [file 40479_2021_176_MOESM1_ESM.docx]

*Development of a German set of qualitative descriptors for pleasant touch perception*

To develop a set of qualitative sensory and affective descriptors of pleasant touch, we were guided by the touch perception task (TPT) from Guest et al. [1]. In a back translation design, all 96 adjectives that were identified to provide a candidate lexicon for sensory and emotional aspects of touch by Guest et al. [1] were translated from English into German. Afterwards they were re-translated into English by a second translator. The original and the back translated version were compared to obtain the final German version. Finally, each adjective was evaluated by both translators to ensure semantic correspondence. Both translators are a bilingual person with German as first language.

In a pilot study we applied pleasant touch to 14 healthy control subjects (6 male) using the same custom apparatus as in the main study. In this study, the touch was applied to the left forearm for one minute in 10 blocks. After each block up to 10 adjectives were presented and the subjects rated to which extend the respective aspect of touch was appropriate on a 5-point Likert scale (“not appropriate” – “completely appropriate”). From all 96 adjectives, we selected for the present study, those adjectives that were (1) included in the final version of the TPT, or (2) defined as low loading in the preliminary version of the TPT by Guest et al. [1]. Additionally we selected those that were (3) of relevance for pleasant touch (defined as rating “not appropriate” from less than 67%) in our pilot study. This procedure results in a final set of 59 adjectives, that were used in the main study (37 sensory, 22 affective) (see table S1).

*Assessment and analysis of qualitative aspects of touch perception*

In the main study, after each block of touch stimulation up to 10 of the 59 selected adjectives were presented on the computer screen. For each adjective, subjects indicated to what extend it was applicable, on a visual analog scale ranging from 0 (“not appropriate”) to 100 (“exactly appropriate”). Each adjective was presented twice, once after a trial with startle and once after a trial without startle.

We calculated the sensory and affective factors of the TPT [1] by the mean of the rating of the respective attributes. For these factors, ratings for trials with and without startle were compared using paired sample t-tests for both groups separately. For group comparisons, t-tests for independent samples were conducted using the TPT factors as dependent variables. We descriptively analyzed the remaining adjectives separately, and we report *M* and *SD* (see table S2).

Table S1: Sensory and affective attributes of pleasant touch

| **English original** | **German translation** |  | **English original** | **German translation** |  | **English original** | **German translation** |  | **English original** | **German translation** |
| --- | --- | --- | --- | --- | --- | --- | --- | --- | --- | --- |
| rough^1a^ | rau |  | firm^1c^ | fest |  | pleasurable^1e,f^ | vergnüglich |  | textured^3^ | strukturiert/ texturiert |
| smooth^1a^ | glatt |  | sharp^1c^ | scharf |  | exciting^1f^ | aufregend |  | velvety^3^ | samtig |
| bumpy^1a^ | uneben |  | hot^1c^ | heiß |  | arousing^1f^ | erregend |  | wooly^3^ | wollig |
| prickly^1a^ | stachlig |  | burning^1c^ | brennend |  | thrilling^1f^ | spannend |  | cool^3^ | kühl |
| soft^1a^ | weich |  | fuzzy^1d^ | flaumig |  | sensual^1f^ | sinnlich |  | blissful^3^ | herrlich |
| lumppy^1a^ | klumpig |  | fluffy^1d^ | flauschig |  | sexy^1f^ | sexy |  | heavenly^3^ | himmlisch |
| gritty^1a^ | kieselig |  | dry^1d^ | trocken |  | hairy^2^ | haarig |  | intense^3^ | intensiv |
| jagged^1a^ | zerklüftet |  | irritating^1e^ | irritierend |  | sticky^2^ | klebrig |  | meaningful^3^ | bedeutungsvoll |
| wet^1b^ | nass |  | comfortable^1e^ | bequem |  | vibrating^2^ | vibrierend |  | nice^3^ | nett |
| damp^1b^ | feucht |  | discomfort^1e^ | unbehaglich |  | warm^2^ | warm |  | weird^3^ | merkwürdig |
| greasy^1b^ | fettig |  | relaxing^1e^ | entspannend |  | feathery^3^ | federig |  | gentle^3^ | behutsam |
| cold^1b^ | kalt |  | calming^1e^ | beruhigend |  | furry^3^ | pelzig |  | tender^3^ | zärtlich |
| slippery^1b^ | rutschig |  | soothing^1e^ | wohltuend |  | satiny^3^ | seidig |  | gummy^4a^ | gummihaft |
| rubbery^1b^ | gummiartig |  | enjoyable^1e,f^ | angenehm |  | silky^3^ | seidenweich |  | spongy^4b^ | schwammartig |
| hard^1c^ | hart |  | desirable^1e,f^ | begehrenswert |  | squishy^3^ | schwammig |  |  |  |

^1^Attributes of the factors of the original version of the touch perception task (TPT, Guest et al., 2010) ^1a^ sensory factor roughness ^1b^ sensory factor slip ^1c^ sensory factor firmness ^1d^ sensory factor pile ^1e^ affective factor comfort ^1f^ affective factor arousal ^2^ low loading attributes in the original version of the TPT ^3^adjectives of relevance for pleasant touch ^4^ >67% rejection in pilot study but used due to it semantic similarity to the German word for rubbery^a^ or squishy^b^

Table S2: Perception of touch in trials with and without startle in patients with borderline personality disorder and healthy controls

|  | **BPD** (n = 25) | | | **HC** (n = 25) | | |
| --- | --- | --- | --- | --- | --- | --- |
|  | **Without startle**  *M* (*SD*)  Mdn (IQR) | **With startle**  *M* (*SD*)  Mdn (IQR) | **statistics** | **Without startle**  *M* (*SD*)  Mdn (IQR) | **With startle**  *M* (*SD*)  Mdn (IQR) | **statistics** |
| **Intensity** | 53.08 (18.13)  54.17 (28.08) | 56.09 (18.31)  58.00 (26.25) | z= -1.06, p= .290 | 74.91 (19.19)  74.83 (29.50) | 74.36 (19.28)  72.67 (30.50) | z=-1.18, p= .247 |
| **Valence** | -4.91 (41.14)  -17.67 (57.00) | -4.09 (43.99)  -6.00 (64.00) | t(24)= -0.22, p= .828 | 57.49 (39.79)  64.67 (51.33) | 56.21 (40.51)  66.67 (53.00) | t(24)= 0.59, p= .562 |
| **Roughness** (TPT) | 27.05 (15.16)  24.50 (24.75) | 28.55 (18.80)  23.88 (30.88) | z= -0.03, p=.976 | 13.68 (10.86)  11.63 (14.19) | 13.29 (12.18)  9.38 (15.06) | z=-0.67, p=.501 |
| **Slip** (TPT) | 13.58 (15.46)  8.67 (22.25) | 14.58 (18.87)  5.17 (27.08) | z= -1.11, p= .268 | 7.77 (9.77)  2.17 (15.33) | 6.67 (10.38)  2.17 (11.08) | z= -0.09, p= .931 |
| **Firmness** (TPT) | 24.3 (16.37)  26.00 (27.60) | 22.58 (16.29)  21.00 (27.30) | t(24)= 0.58, p= .565 | 9.61 (12.73)  5.60 (12.60) | 8.69 (11.45)  6.20 (10.80) | z= -0.11, p= .914 |
| **Pile** (TPT) | 46.2 (19.70)  46.00 (29.33) | 48.05 (22.75)  52.67 (32.67) | t(24)= -0.60, p= .554 | 55.84 (27.18)  61.67 (42.00) | 58.89 (26.64)  66.00 (34.83) | z= -0.61, p= .539 |
| **Comfort** (TPT) | 36.75 (19.00)  40.89 (33.39) | 36.87 (22.20)  32.22 (30.44) | t(24)= -0.05, p= .958 | 66.06 (20.39)  72.56 (21.11) | 62.38 (20.03)  64.11 (23.56) | t(24)= 2.54, p= .018 |
| **Arousal** (TPT) | 20.9 (17.73)  16.50 (28.81) | 24.55 (17.74)  22.25 (32.69) | t(24)= -1.80, p= .084 | 36.09 (21.94)  35.63 (31.00) | 34.8 (19.15)  35.88 (27.69) | t(24)= 0.79, p= .436 |

BPD = Borderline personality disorder; HC = healthy control; n = number; *M* = mean; *SD* = standard deviation; TPT = Touch perception task

*Results on other sensory modalities*

Mechanical detection threshold (MDT) was significantly higher in BPD (*Mdn* = 2.645) compared to HC (*Mdn* = 1.28), *U* = 155.5; *z* = -3.05, *p* = .002, *r* = .43, indicating a reduced touch sensitivity in BPD compared to HC.

There was no significant difference in warm perception thresholds between the BPD (*Mdn* = 34.29) and HC groups (*Mdn* = 34.04), *U* = 134.00, z = -1.20, *p* = .230, *r* = .19. Descriptively heat pain threshold in BPD (M = 46.75°C, SD = 3.21) was higher than in HC (M = 44.40°C, SD = 3.93). But the difference did not reach significance level, *t*(36) = 1.896 *p* = .066, *d* =.54.

In the HC group, there was a significant positive correlation between HPT and perceived valence of touch (*r*(22) = .456, p = .025), indicating that a more positive perception of touch was associated with a higher pain threshold. There was no significant correlation between HPT and perceived intensity of touch in HC (*r_s_*(22) = -.188, *p* = .379) or between HPT and perceived valence (*r*(12) = -.183, *p* = .531) or intensity of touch (*r_s_*(12) = .007, *p* = .982) in BPD.

*Body ownership experiences and state dissociation in HC*

Table S3: Body ownership and state dissociation before and after stimulation with pleasant touch in HC

|  | **Pre**  *M* (*SD*)  *Mdn (IQR)* | **Post**  *M* (*SD*)  *Mdn (IQR)* | **Change**  *M* (*SD*)  *Mdn (IQR)* |
| --- | --- | --- | --- |
| **Body ownership**  **stimulated left arm [%]** | 99.66 (1.17)  100.00 (0.00) | 99.32 (2.34)  100.00 (0.00) | -0.34 (1.60)  0.00 (0.00) |
| **Body ownership**  **non stimulated right arm [%]** | 99.89 (0.53)  100.00 (0.00) | 99.77 (1.07)  100.00 (0.00) | -0.11 (1.21)  0.00 (0.00) |
| **State dissociation**  **(DSS-4)** | 0.16 (0.40)  0.00 (0.00) | 0.11 (0.25)  0.00 (0.00) | -0.05 (0.22)  0.00 (0.00) |

Pre = before pleasant touch application; Post = after pleasant touch application, Change = Post-Pre; *M* = mean; *SD* = standard deviation; *Mdn* = median; *IQR* = interquartile range; DSS-4 = Short version of the Dissociation tension scale acute [2]

**References**

[1] Guest S, Dessirier JM, Mehrabyan A, McGlone F, Essick G, Gescheider G, et al. The development and validation of sensory and emotional scales of touch perception. Attention, Perception, Psychophys 2011;73:531–50. https://doi.org/10.3758/s13414-010-0037-y.

[2] Stiglmayr C, Schmahl C, Bremner JD, Bohus M, Ebner-Priemer U. Development and Psychometric Characteristics of the DSS-4 as a Short Instrument to Assess Dissociative Experience during Neuropsychological Experiments. Psychopathology 2009;42:370–4. https://doi.org/10.1159/000236908.
